# Apraxia: From Neuroanatomical Pathways to Clinical Manifestations

**DOI:** 10.1007/s11910-024-01391-6

**Published:** 2024-11-16

**Authors:** Sarah Stoll, Lukas Lorentz, Ferdinand Binkofski, Jennifer Randerath

**Affiliations:** 1 Department of Psychology, University of Konstanz, Konstanz, Germany; 2 Lurija Institute for Rehabilitation Science and Health Research, Kliniken Schmieder, Allensbach, Germany; 3 Department of Developmental and Educational Psychology, Faculty of Psychology, University of Vienna, Vienna, Austria; 4 Division for Clinical Cognitive Sciences, Department of Neurology, University Hospital RWTH Aachen, Aachen, Germany; 5 Clinical Neuropsychology and Neuropsychological Psychotherapy, Institute of Psychology, University of Regensburg, Regensburg, Germany

**Keywords:** Apraxia, Tool use, Gestures, Pathways, Model, Digital

## Abstract

**Purpose of Review:**

Apraxia typically involves impairments in gesture production and tool use, affecting daily life activities. This article reviews current conceptualizations and developments in diagnostic and therapy.

**Recent Findings:**

Apraxia has been studied in various neurological conditions, particularly stroke and dementia, but recent studies show gesturing deficits in psychiatric populations as well. Promising results have emerged from integrative treatment approaches involving intensive practice of gestures or daily activities. However, several reviews have noted the only marginal progress in apraxia therapy research despite new technologies, like virtual reality and brain stimulation, offering fresh opportunities for assessment and therapy.

**Summary:**

Advances in lesion-symptom mapping and connectivity analyses led to more detailed neuroanatomical models emphasizing parallel and gradual processing. These models facilitate the understanding of underlying mechanisms of motor cognitive performance and its decline. Finally, the digital era prompts the need to study digital tool use in apraxia, with initial efforts underway.

## Introduction

Daily, we engage in numerous manual activities, whether it’s manipulating tools and objects or using gestures for communication purposes. Various studies using different Neuroimaging methods suggest the existence of different pathways and central hubs within networks of the primate brain that are involved while planning actions and interacting with objects in the environment [1–5]. These neural pathways, central hubs, and connections are thought to be crucial in specifying, selecting, and integrating pertinent information to execute actions [6–9]. Damage to critical nodes or pathways in this praxis network due to conditions like stroke, traumatic brain injury, or degenerative diseases such as Alzheimer’s can lead to disruption in neuropsychological functioning, resulting in difficulties with motorcognitive tasks. This disruption may manifest as challenges in tasks involving imitating meaningful or meaningless hand postures, selecting appropriate tools and objects for specific purposes, grasping tools efficiently, utilizing tools and objects correctly, deducing the function of novel tools, monitoring action sequences, and producing communicative gestures. This conglomerate of motor cognitive impairments is commonly coined with the term (limb) apraxia. Apraxia cannot be primarily attributed to muscle weakness, akinesia, sensory differentiation issues, abnormal muscle tone or posture, movement disorders like tremor or chorea, intellectual decline, poor comprehension, or lack of cooperation [10]. While apraxia is most frequently observed and severe in cases of left-brain damage or dementia, it can arise from numerous neurological or psychiatric conditions [11–17]. In patients with left hemisphere stroke limb apraxia is evident in approximately 50%. The disorder amplifies disability and caregiver dependence. For limb apraxia, the complexity of the disorder, reflected by its many facets, has presented a major challenge for classifications and therapy.

## Clinical Manifestations and Approaches for Assessment and Therapy

At the beginning of the 20th century, the German neurologist K.H. Liepmann laid the foundation for the study of apraxia by publishing a series of classical studies with groups of brain-damaged patients (e.g., [18]). Typical errors in limb apraxia include movements that appear inaccurate and clumsy. When asked to imitate a hand posture, patients can often not achieve the demonstrated spatial position of the hand. When asked to pantomime a typical action, e.g., how to hammer, patients often appear clueless and produce incorrect or incomplete movements. Also, while interacting with real objects, patients may demonstrate conceptual or content-related problems, such as scrubbing a mirror with a toothbrush instead of brushing one’s teeth. Further, they may show difficulties in performing multi-step actions, such as omitting aspects or reversing the order of steps. For example, switching on the toaster but omitting to insert bread, or operating the kettle without water. Liepmann classified these error types into limb kinetic, ideomotor, and ideational apraxia [18, pp. 524]. However, we refrain from expanding on these categories due to inconsistencies in their use and interpretation (for a more detailed description see e.g. [19]). Instead, to ensure a consistent understanding amongst researchers and clinicians, we recommend following Goldenberg [20] to define the impaired aspects of limb apraxia based on describing the patient’s performance in the assessed subtests and behavioral observations.

Identifying limb apraxia can be difficult because aphasia, memory problems, or hemiplegia can cooccur and mask the apraxia deficits. To confirm that the patients understood the task and that the demonstrated problems cannot be explained by motor-related problems (i.e. hemiplegia), most tests begin with simpler items, and it is recommended that the ipsilesional hand is tested. Depending on the situation, screenings or comprehensive instruments are best suited to assess the syndrome of limb apraxia (for an overview see [21]). Various subtests, each lasting between 2 and 15 min, cover different apraxia symptoms. Most contemporary studies and diagnostic instruments focus on gesture production or imitation, while movement coordination, real tool use, or multistep actions have rarely been addressed. Tasks like rotating a coin between the fingers can assess movement coordination (e.g. [22]), or sensors can be applied to obtain kinematic variables characterizing movement fluency, velocity, and precision while performing different actions (e.g. [23, 24]). Impaired gesture production is typically assessed by subtests evaluating the ability to imitate meaningless or meaningful gestures and to pantomime the use of tools. These classic gestural tests can vary in whether the movements have to be demonstrated based on verbal command alone or verbal command supported by visual information such as a pictured object. The latter provides two modes of perceptual access and can help secure task comprehension. Also, for assessing tool use, several distinctions need to be made. Differences in performance and dissociations have been demonstrated, for example, depending on whether only the tool (demonstration of tool use) or also the recipient object to be manipulated (real tool use) are presented [25, 26]. Further, for real tool use, a distinction is made between the ability to select versus applying tools and between novel versus familiar tools and objects [27, 28]. It is assumed that mechanical problem-solving is crucial for handling novel tools, whereas, for the use of familiar objects, the retrieval and integration of semantic knowledge into an action plan are particularly important [29]. Multi-step tasks, such as preparing breakfast, allow for the evaluation of more complex actions and their sequencing in rather cluttered environments [30, 31]. Next to difficulties with producing skillful movements, problems may also occur in recognizing suitable actions [32] or in a lack of awareness of their apraxic deficits [33, 34]. It needs to be noted that frequently, different subtests can be affected in one individual.

Regarding interventions, gesture training and strategy training of everyday activities, are the best studied. The gesture training by Smania, Aglioti [35]consists of exercises for gesture production with a total of 35 approximately 1-hour sessions. Training is carried out with cues of decreasing salience, such as demonstrating the actual execution, the presentation of a pantomime, or an illustration. In some patients who received training, improvements were observed even after 2 months. An adapted and shorter version implementing the demonstration of tool use was successfully applied in a single-case pilot study by Stoll, de Wit [36]. The strategy training, according to Geusgens, Van Heugten [37], targets activities in daily living and considers the needs of the affected individuals in their specific environment. Participants select and train activities that they consider important. The individual’s problems due to apraxia are identified, and specific strategies are used to train the tasks (e.g., self-verbalization of procedures: first I unscrew the cap from the toothpaste tube, then...). The strategy training led to improvements in trained and non-trained everyday activities, which were also maintained after 5 months. A similar approach, the Naturalistic Action therapy, piloted by Buchmann, Finkel [38] highlighted the importance of patient self-evaluation and therapist feedback on both deficits and successes while exercising the tasks [39].

## Apraxia Models

As early as the beginning of the 20th century, neuroanatomical models for apraxia tried to ascribe disturbed motor cognitive components to damaged brain regions [15, 18, 40]. These models were mostly informed by post-mortem analyses of the patient’s brains. Later, the complex nature of limb apraxia was displayed by cognitive models that connected functional aspects of perception and action [41, 42]. The disentanglement of the many components involved in motor cognitive performance was nourished by observed behavioral dissociations in single patients. Perhaps the most influential cognitive neuropsychological model of limb apraxia was published by Rothi, Ochipa [42]. It has three key modules. First, Multimodal Input Systems analyze incoming stimuli (e.g., visual or auditory). From here, there is access to conceptual systems (e.g., pantomime of tool use) or a direct translation from input into output patterns (e.g., imitating meaningless gestures). Second, Action Memory and Semantics Systems are important for familiar actions like tool use, enabling object and action recognition and production. They also store conceptual knowledge about tools, objects, and communicative gestures. Third, direct Translation from Visual to Movement Patterns allows for the production of novel actions. Current adapted models (e.g. [19]) pronounce the importance of processing visuospatial relationships on this route, which is thought to be crucial for novel tool use and meaningless gesture imitation [43]. In addition, an action working memory system has been proposed that integrates and maintains processed information from these routes [44–46].

Over the past decade, lesion-symptom mapping approaches have contributed significantly to identifying essential brain regions associated with performance in tasks commonly used to assess apraxia [47]. For example, poorer performance in the imitation of meaningless gestures is often associated with lesions primarily in left parietal areas [27]. A study on stroke patients with left-hemispheric damage compared error types in pantomime [48]. The results showed differently associated lesion areas for communicative aspects (inferior frontal) versus motorcognitive aspects (inferior parietal) for impaired pantomime execution. A recent study on left-hemispheric stroke [27] showed a difference in lesion maps between tool types. While impaired selection of familiar tools was associated with damage in more ventral brain areas (frontotemporal), impaired selection of novel tools showed more dorsal lesion maps (frontoparietal). A similar pattern emerged for movement execution, although lesion maps for impaired use of novel tools and familiar tools overlapped considerably in inferior frontal and parietal areas. Further, studies indicate that the successful execution of multi-step actions can be hindered by brain damage in a variety of locations [49]. As the title of a study by Hartmann, Goldenberg [50] aptly describes, “It takes the whole brain to make a cup of coffee.” Whether the effect of lesions in the frontal cortex is more pronounced during multistep actions with tools and objects as compared to single-step tool use still needs to be studied in more detail. In the past two decades a range of apraxia lesion studies have carved out a predominantly left hemisphere network to be important in persons with right-hand dominance [51–53].

**Fig. 1:**
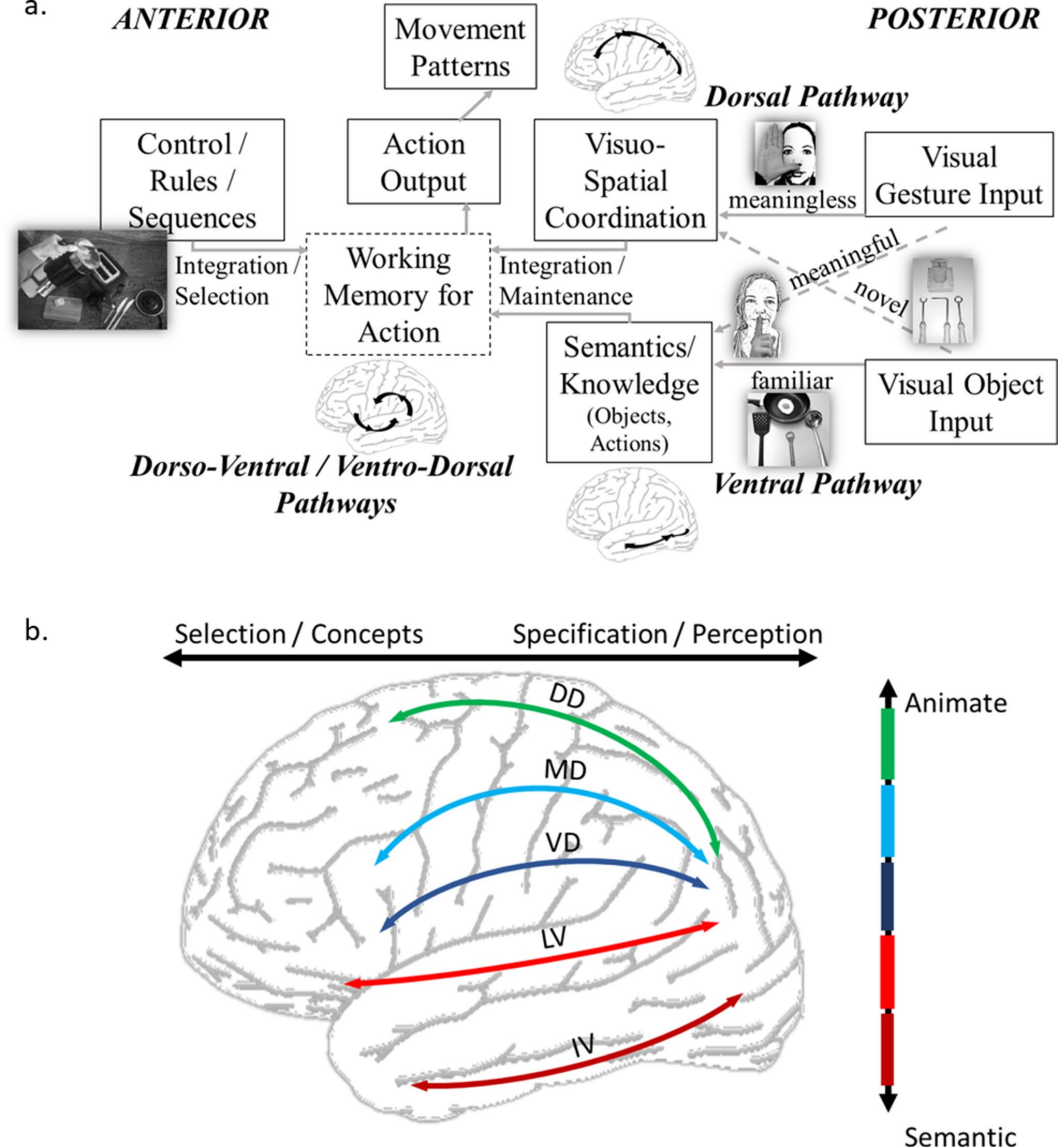
(**a**) Simplified model of limb apraxia including cognitive (boxes) and neuroanatomical (bold) aspects as well as typically applied subtests (pictured) that are thought to assess the apraxic aspects. (adapted from 9). 1(**b**): Comprehensive model of sub-parcellation of dorsal and ventral streams. (DD– dorso-dorsal stream; MD– media-dorsal stream; VD– ventro-dorsal stream; LV– latero-ventral stream; IV– infero-ventral stream). Whereas DD is processing pure motor information, is IV processing pure semantic information. In between there is a gradient of animate motor information from dorsal to ventral and a gradient of semantic information from ventral to dorsal. The sub-streams act in parallel and are used in combination as required by the actual action demands. 1a and b: To interact with tools and objects in the environment, an action selection network is proposed to be predominantly located in anterior regions, and an action specification network primarily in posterior regions. The motorcognitive network is believed to function alongside integrative working memory hubs and, as part of a dynamic system, it is assumed to undergo updates through recurrent perception-action loops (see, for instance [55])

At the same time, several rather overarching neuroanatomical models have been proposed to explain motorcognitive abilities in the healthy brain [6, 54–56]. Assumptions of dynamic and parallel processing have replaced the previously assumed hierarchical processes between information uptake and action output [19, 43]. Accordingly, our assumptions on how the brain tackles interactions with the environment, such as tool use, draw from explanatory accounts with a unifying perspective coupling action and perception reciprocally rather than assuming a one-way perception-action hierarchy. For example, three such theoretical approaches have been influential: the theory of event coding, the affordance competition hypothesis, and the theory of active inference. The theory of event coding hypothesis proposes that perception and action are interconnected processes operating on the same “event files,” which temporarily bind features during an event. Later, encountering similar features or information can trigger the retrieval of these event files, aiding recognition and action [54, 57]. The affordance competition hypothesis posits that the brain simultaneously considers multiple potential actions (affordances) in a dynamic, competitive process [6]. The selection of action is influenced by sensory input, internal goals, context, and learned biases. As the brain continuously evaluates and updates the possible actions based on ongoing sensory information and goals, the actions or motor commands are simultaneously specified. The theory of active inference emphasizes the brain’s use of internal models to predict and direct actions [56]. Here, the brain actively anticipates events and updates its internal models through actions, minimizing uncertainty and surprise [58]. The apraxia research community has increasingly drawn from unifying perspectives and neuroanatomical perception-action pathway models to advance their understanding of its underlying mechanisms [1, 9, 59]. The apraxia model depicted in Fig. 1 integrates in a simplified manner cognitive and neuroanatomical aspects.

## Latest Advances in the Conceptualization of Apraxia

Limb apraxia is a disorder at the intersection of motor control and cognition reflected by lesions in ventro-dorsal pathways, with a particular role of the inferior parietal lobe [43]. We here propose that the syndrome of apraxia should be looked at as a range of symptoms on a continuum between perception, motor control, and cognition reflected by lesions on the continuum of ventral to dorsal and posterior to anterior reciprocal pathways. The idea that apraxia should be viewed as a continuum dates back to the beginning of the 20th century. Liepmann’s neuroanatomical model of apraxia proposed a continuum from problems with the more basic motor to more complex cognitive aspects of limb apraxia reflected by respective lesion sites at the transition from dorsal to ventral regions [15, 18, 60]. Thus, in principle, Liepmann’s model and assumptions have surprising timeliness. The last decades have primarily focused on the midst of this continuum, apraxic behavior in gesture production represented by lesions in the center of the praxis network. These highlight the role of a ventro-dorsal stream and the inferior parietal lobe as an integrative hub for motor cognitive aspects [19] (see Fig. 1). Less attention has been paid to the edges of Liepmann’s continuum: the motor side or the executive control, respectively. On the motor side, these are clumsy fine motor skills resulting in difficulties producing individual limb movements. These symptoms are supposedly associated with more dorsal lesion sites in close proximity to motor regions [19]. Thus far, disentangling motor aspects from rather conceptual problems has been primarily addressed by experimental approaches (e.g., studies including kinematics or grip force measures) rather than standardized assessments. For example, despite comparable performance in a tea-making task, Gulde, Hughes [61] could detect kinematic features that differed between left-hemisphere stroke patients and healthy control participants. At the other end of Liepmann’s continuum, there are problems with executing multistep actions in need of executive control. Here, in contrast to Liepmann’s assumptions, we expect more anterior and frontal lesion sites [19]. Clinical assessments evaluating multi-step actions may have to better disentangle the evaluation of motor and executive function aspects since both contribute to erroneous behavior while interacting with tools and objects during sequential actions.

Current neuroanatomical models of apraxia have benefited from pathway models that outline the flow of information from perception to action. Based on animal data, the classic model of visual information processing by Ungerleider and Mishkin [62], identified two cortical visual systems: a dorsal stream starting in the primary visual cortex (V1) and leading to the posterior parietal cortex (PPC) for spatial perception (“where” pathways) and a ventral stream connecting to the inferior temporal cortex (IT) for object recognition (“what” pathway). Goodale and Milner [4] refined this model using human patient data. They showed that patients with ventral stream damage could still grasp objects despite being unable to recognize them (agnosia), while those with dorsal stream damage could recognize objects but struggled with accurate hand movements (optic ataxia). While agreeing that the ventral stream handles object recognition, they proposed that the dorsal stream is vital for the real-time visual guidance of actions, redefining it from “where” to “how” and disregarding its spatial role.

Supported by modern neuroimaging techniques, the influential dual pathway model has been extensively tested, discussed, and extended in the past thirty years (see Fig. 1).

**The dorsal system and its three substreams.** Rizzolatti and Matelli [2] showed that the dorsal stream consists of two parts: the dorso-dorsal stream, which runs through the superior parietal lobule and controls real-time actions, and the ventro-dorsal stream, which organizes actions and handles space perception and action understanding. They reciprocally proposed that motor activity provides knowledge useful for perception. Building on this, Binkofski and Buxbaum [1] labeled the dorso-dorsal stream the “Grasp” system, managing object shape and size for quick actions, and the ventro-dorsal stream the “Use” system, storing skilled actions for familiar objects. Lesions in these streams show distinct impairments—ventro-dorsal damage is linked to limb apraxia and likely to affect tool use [27, 47, 63], while dorso-dorsal damage can lead to optic ataxia and problems in reaching and grasping [64–66]. For example, Randerath, Goldenberg [5] demonstrated that patients who showed erroneous grasping of tools according to their function had prominent lesions in the inferior frontal gyrus (IFG) and the angular gyrus (ANG), whereas impairment in tool-use was predominantly associated with damage in the supramarginal gyrus (SMG). A third left-lateralized pathway, first described by Jeannerod, Arbib [67] in macaques, links the IPS area aIP with the ventral premotor area F5 and is dedicated to hand-object interaction. In a recent study Jüchtern, Shaikh [68] found structural evidence for a third medio-dorsal stream in humans using high-resolution DTI analysis of parieto-premotor connectivity. These findings are supported by a previous fMRI study by Binkofski, Buccino [69], which found that aIPS and the ventral premotor cortex facilitate fast and efficient object and tool interactions.

A key question is whether the parietal counterpart of this network in humans is the area AIP or the neighboring aSMG. Orban and Caruana [70] suggested that aSMG evolved from AIP to accommodate humans’ advanced object and tool use, making it a counterpart to ventral premotor areas. While DTI studies offer precise anatomical data, evidence from lesion studies may reach its boundaries with further segregations. Stoll, Finkel [27] found that lesions in the medio-dorsal system were linked to poor performance with novel tool use, suggesting this fronto-parietal system aids in action simulation. The SMG was crucial for action execution, involving both novel and familiar tool use and gesture imitation and was therefore interpreted as essential for appropriate action specification.

**The ventral system and its two substreams.** In their review Wurm and Caramazza [71] suggest two parallel sub-streams for the ventral stream: a latero-ventral sub-stream for action recognition, processing both semantic and kinematic information, and an infero-ventral sub-stream focused on pure semantic data related to object categories and identity. This forms a dorsal-ventral distinction for animate and inanimate action aspects. They also identify a posterior-anterior gradient, moving from perceptual to conceptual representations.

**Substreams and gradual changing functional dominance.** By having a closer look at the new sub-parcellation of the dorsal and the ventral streams we can propose a unified model of parallel processing of visuo-motor information in the two streams with a ventral to dorsal gradient: the dorsal stream focuses more on action, while the ventral stream handles more semantic processing, though both streams overlap. The dorso-dorsal sub-stream handles pure action, and the infero-ventral sub-stream focuses on pure semantics, with varying degrees in between. This framework can explain the manifold proposed dorsal-ventral segregations between lesion maps associated with different functions, such as impairments in tool selection for novel versus familiar tool use [27], or manipulation versus functional action knowledge [72, 73]. Moreover, the assumption of gradually changing dominance of functions across the streams helps to understand discrepancies between the interpretation of lesion study results in the realm of apraxia as these may depend on slight differences in the assessed tasks and aspects [53]. This framework aligns with Ungerleider and Mishkin [62] classic “where” (dorso-dorsal) and “what” (infero-ventral) streams while incorporating novel developments.

## Current Developments in Apraxia Assessment and Therapy

In the last few years, gestural deficits have been reported in several other patient groups suffering from neurodegenerative or psychiatric disorders. Deficits in gesture imitation and/or pantomime tasks are reported in neurological populations, such as patients with corticobasal syndrome [74, 75], Richardson’s syndrome [76], Parkinson’s disease [77], Alzheimer’s disease [78], and multiple sclerosis [79]. Also in psychiatric populations, deficits in imitating gestures and pantomiming were observed, such as in patients with major depressive disorder [80] and patients with bipolar disorder (type I) [81]. There is a growing field of research on apraxia in patients with schizophrenia, reporting its prevalence and clinical correlates [82, 83], and proposing underlying mechanisms, e.g., reduced efficiency of praxis network, reduced integrity of the longitudinal and arcuate fasciculi, and the corpus callosum [84]. Thus far, these studies concentrated on gestural deficits, except for one reporting means and standard deviations also for actual object use in patients with schizophrenia and bipolar disorder [81]. The apraxia profiles indicating potentially differentially affected subtests across the different psychiatric populations still need to be determined. This may be because assessments that consider real tool use [30, 31] emerged more recently and are typically affected in fewer cases. However, difficulties in selecting the adequate tool and correctly applying it have been reported in patients with dementia and left hemisphere stroke and can considerably affect independence in activities of daily living [12, 27, 28].

Additionally to “traditional” tools (e.g., kitchen utensils, office gadgets), smart digital devices became *the* tool of the 21st century [85]. However, there are currently hardly any attempts to assess digital competencies. The newly developed “Digital Tools Test” (“DIGI”) [86, 87] includes everyday tasks that must be solved with a smartphone or tablet. It allows evaluation of the correct app selection, app use, and whether motor-related errors occurred. Thus far, its feasibility has been proven in young and older adults [87]. Interestingly, digital competencies correlated with the proficiency to use novel tools only in the older sample. A pilot study applying the DIGI in neurological patients is ongoing.

Research on limb apraxia started to consider more technical approaches such as brain stimulation techniques, which may strengthen training effects, or virtual and augmented reality environments, which may offer new compensating strategies and a safe opportunity to create more complex training environments. For instance, augmented reality settings using three-dimensional holographic cues improved the pantomime of tool use compared to two-dimensional cues [88]. Virtual reality approaches yielded promising results regarding improvements in imitation [79] and pantomime of gestures [89]. Despite such promising findings, there remain concerns regarding the transferability of virtual training to the physical world [90] or the detection of clinical symptoms of apraxia by the use of virtual environments [91]. As technology advances, these methods must be constantly reevaluated, and their possible applications in diagnosis and therapy may be extended. For the treatment of apraxia, stimulation techniques are currently discussed and applied in different populations. Inhibitory approaches (e.g., administering transcranial continuous theta burst stimulation) on the right inferior parietal cortex reduce its inhibiting effect on its homolog area in the left hemisphere, leading to improvements in gesturing in patients with left-hemisphere stroke [92] and with schizophrenia [93]. Complementary, the facilitating stimulation of the left inferior frontal gyrus [93] also improved gesturing in schizophrenia patients. The facilitating effects of anodal direct current stimulation further improved sequential buttoning and unbuttoning, in patients with Parkinson’s disease [94]. Technical approaches such as augmented or virtual environments and stimulation techniques such as tDCS or TMS have made a promising launch in limb apraxia. With all the advantages and advances of technical approaches, we consider it important to also strive for broadening training content and elevating training studies from the pilot phase to controlled clinical trials.

## Conclusions

For a long time, studies investigating gesture production dominated the field. In recent years, patients’ problems using traditional familiar or novel tool use have been addressed more thoroughly. Now, in the era of digitalization, there is a need to address digital tool use and similarly develop and apply specific assessments and training and study the underlying constructs. The first steps are being made. Several reviews in the past 20 years have mourned the lack of therapy studies. Some promising results have been reported by studies with rather small samples, including stimulation of the brain, technical devices as support, virtual reality, or integrative behavioral therapy approaches. But unfortunately, this manuscript has to join the lamentation about the lack of clinical trials in this field. Some advances have been made in fundamental research, especially in lesion-symptom mapping leading to more fine-grained neuroanatomical models. The role of regions of interest and connectivity analyses in the injured brain, making use of different qualitative and quantitative techniques carving out the differentiation between primarily executive, conceptual, or kinematic problems next to the role of integrative hubs will further the advancements in unraveling the syndrome of limb apraxia. These developments go along with reported incidences of apraxia not only in neurological but also in psychiatric populations, underlining the importance of advances in limb apraxia neurorehabilitation.

## Key References

- Wurm MF, Caramazza A. Two ‘what’pathways for action and object recognition. Trends in cognitive sciences. 2022;26 [2]:103 − 16. 10.1016/j.tics.2021.10.003.
  This article distinguishes two separate “what” pathways within the ventral visual stream: one originating in the lateral occipito-temporal cortex (LOTC) primarily involved in action recognition and another mainly located in the ventral occipito-temporal cortex (VOTC) for object recognition. Within the lateral action pathway, there is a posterior–anterior gradient from perceptual action precursors to conceptual action representations and a dorsal–ventral distinction that segregates animate and inanimate action aspects. This distinction explains several phenomena of functional organization and concretizes the role of the LOTC in apraxia.
- Jüchtern M, Shaikh UJ, Caspers S, Binkofski F. A gradient of hemisphere-specific dorsal to ventral processing routes in parieto-premotor networks. Network Neuroscience. 2024:1–63. 10.1162/netn_a_00407.
  Description of a third dorsal visuomotor pathway for information processing. Besides the Dorso-Dorsal Stream and the Ventro-Dorsal Stream there is a third Medio-Dorsal Stream. The Medio-Dorsal Stream is dedicated to direct hand-object interaction. This new triadic division helps to explain the apraxic deficits much better.
- Stoll SE, Bauer I, Hopfer K, Lamberty J, Lunz V, Guzmán Bausch F, et al. Diagnosing homo digitalis: towards a standardized assessment for digital tool competencies. Frontiers in psychology. 2024;14:1270437. 10.3389/fpsyg.2023.1270437.
  Clinical observations revealed that patients with apraxia not only demonstrate difficulties in using common tools, but also when handling digital tools, like smartphones. The advancement of digital tool competency assessments and accordingly rehabilitation is essential when we aim at facilitating societal inclusion and participation for individuals with apraxia. The study by Stoll et al. introduces the Digital Tools Test (DIGI), a novel assessment aimed at evaluating common digital tool competencies.

## Data Availability

No datasets were generated or analysed during the current study.
